# Measurement of the ankle brachial index with a non-mercury sphygmomanometer in diabetic patients: a concordance study

**DOI:** 10.1186/1471-2261-13-15

**Published:** 2013-03-08

**Authors:** Magdalena Bundó, Magali Urrea, Laura Muñoz-Ortíz, Carmen Pérez, Judit Llussà, Rosa Forés, María Teresa Alzamora, Pere Torán

**Affiliations:** 1 Primary Health Care Centre Ronda Prim, Catalan Health Institute, Camí del Mig 36, (4th floor), Mataró, Barcelona, 08330, Spain; 2 Primary Health Care Research Support Unit Metropolitana Nord, IDIAP Jordi Gol, Catalan Health Institute, Carrer Major 49-53 (1st floor), Santa Coloma de Gramanet, 08921, Spain; 3 Primary Health Care Centre Llefià, Catalan Health Institute, Carretera Antigua de Valencia s/n, Badalona, Spain; 4 Primary Health Care Centre Sant Roc, Catalan Health Institute, C/ Velez Rubio s/n, Badalona, 08918, Spain; 5 Primary Health Care Centre Riu Nord-Riu Sud, Catalan Health Institute, c/ Major 49 Sta. Coloma de Gramenet, Barcelona, 08921, Spain; 6 Primary Health Care Centre Gatassa , Catalan Health Institute, Camí del Mig 36 (3rd floor), Mataró, 08303, Spain

**Keywords:** Ankle brachial blood pressure index, Peripheral arterial disease, Blood pressure, Type 2 diabetes mellitus, Doppler, Sensitivity and specificity

## Abstract

**Background:**

The removal of mercury sphygmomanometers from health centers requires the validation of other instruments to measure blood pressure in the limbs to calculate the ankle-brachial index (ABI).

**Methods:**

Descriptive cross-sectional study of agreement between two measurement methods in type 2 diabetes patients from three urban primary healthcare centres in the Barcelonès Nord i Maresme area (Catalonia, Spain).

ABI was determined with Doppler and mercury sphygmomanometer and Doppler and the “hybrid” sphygmomanometer OMRON HEM-907 model. Agreement was evaluated using the weighted kappa index. Sensitivity, specificity, positive predictive value (PPV) and negative predictive value (NPV) were calculated using the mercury sphygmomanometer as the gold standard.

**Results:**

211 patients were included, from these, 421 limbs were available for study. The mean age of the participants was 67 years (SD = 10), 51.7% were women.

The index of agreement between ABI measured with a mercury sphygmomanometer and with the OMRON HEM-907 blood pressure monitor was good (weighted kappa index = 0.68; CI 95%: [0.55–0.79]) and improved when the ABI cut-off value was set at ≤0.70 (weighted kappa index = 0.92; CI 95%: [0.81–1.00]). Sensitivity and specificity were 77.5% and 98.2%, respectively. PPV was 83.8% and NPV was 97.3%. With the ABI cut-off value ≤0.70, sensitivity and specificity increased to 85.7% and 100%, respectively, PPV to 100% and NPV to 99.4%.

**Conclusion:**

The combination of a Doppler device with the hybrid sphygmomanometer is a simple and reliable method to measure ABI showing that hybrid sphygmomanometer is a good alternative to the use of mercury sphygmomanometers.

## Background

Peripheral arterial disease (PAD), a clinical manifestation of arteriosclerosis, is associated with increased cardiovascular risk [1,2]. Early detection of PAD, preferably in the pre-clinical stages, is essential to prevent the complications associated with this condition. The ankle-brachial index (ABI) is a simple and non-expensive method to diagnose PAD compared with angiography [3] with high sensitivity and specificity. ABI is calculated by dividing the higher systolic blood pressure (SBP) measured in the ankle by the higher brachial SBP [1]. Many studies have shown the value of ABI as a diagnostic tool for PAD and as an indicator of cardiovascular risk [4,5].

The removal of mercury sphygmomanometers from health care centres based on environmental grounds [6] has prompted to search alternative reliable instruments to measure blood pressure (BP) [7]. Aneroid sphygmomanometers use the auscultatory technique and their accuracy greatly varies from one manufacturer to the other. Also, they require frequent calibration [8].

Oscillometric sphygmomanometers use oscillometric measurements and electronic calculation rather than auscultation. They are validated to determine BP in the arm but there are some limitations in cases of arterial calcification as in the elderly and diabetic patients [8]. To determine ABI, studies have initially excluded patients with arterial calcification in lower limbs otherwise have obtained low sensitivity results [9-12].

Hybrid sphygmomanometers combine features of both electronic and auscultatory devices [8]. The mercury manometer is replaced by a digital display similar to the oscillometric devices. Systolic and diastolic blood pressures are identified by the Korotkoff sounds of the auscultatory technique. These sphygmomanometers have shown efficacy to determine BP and could be the best choice compared to the mercury ones [13,14]. HEM-907 is a model manufactured by OMRON. It performs as a hybrid sphygmomanometer in the manual position and the pressure applied to the limb is continuously indicated. This monitor has been validated for BP measurement in the arm [15], but the applicability of hybrid sphygmomanometers in BP measurement in the lower limbs has not been established.

### Objective

The aim of this study is to determine the agreement between the ABI measured with Doppler and mercury sphygmomanometer and the ABI measured with Doppler and the OMRON HEM-907 hybrid sphygmomanometer.

## Methods

The study took place in three urban Primary Health Care centres in the Barcelonès Nord and Maresme area (Catalonia, Spain) from June to September 2008.

Patients aged 50 or over with type 2 diabetes mellitus were included in the study. The study was approved by the Ethics Committee and all patients signed the consent form. The exclusion criteria were cardiac arrhythmia, a history of PAD surgery, ankle circumference over 40 cm and refusal to sign the informed consent form.

The rationale for the selection of type 2 diabetic patients was their high prevalence of PAD and stiffness of the arteries associated with extreme ABI values [16].

The OMRON HEM-907 hybrid sphygmomanometer determines BP through oscilometric or auscultatory methods according to the choice of the evaluator. For de manual auscultatory BP measurement the cuff inflates automatically and deflates slowly. The pressure applied to the limb is continuously indicated and using the Doppler is possible to determine the SBP through auscultation.

The participants were examined in a relaxed atmosphere with a pleasant temperature. After 10 minute rest in supine position, SBP in the brachial artery was measured in both arms. SBP in the left and right posterior tibial and dorsalis pedis arteries was measured with the cuff placed proximal to the malleoli and the contour adjusted to the conical shape of the lower leg [17].

An 8-mHz Doppler device (Dopplex HNE) was used either with a mercury sphygmomanometer or the OMRON HEM-907 hybrid sphygmomanometer in the manual position. Pulse was located with the Doppler vascular probe and the cuff inflated to restrict blood flow. The examiner slowly released the pressure in the cuff to determine at what pressure blood flow was just starting, and at what pressure it was unimpeded.

In the ankle the SBP was initially measured at the dorsalis pedis pulse following the posterior tibial. If the difference was higher than 10 mmHg a new measurement was done. The new values were recorded if the difference was lower than 10 mmHg. If the difference of 10 mmHg continued a third measurement was done and recorded. The time between measurements was 3 minutes.

These measurements were carried out by two expert health workers especially trained for this study.

Firstly, one of the professionals made the measurements with one of the BP monitors randomly selected. Afterwards, a different healthcare professional blinded to the values obtained in the first examination carried out the measurements in the same patient with the other type of sphygmomanometer. The ABI of each leg was calculated separately by dividing the higher of the two ankle systolic pressures in that leg by the higher brachial systolic pressure value.

The two lower limbs of each patient were considered independent. Therefore, each patient had two posterior tibial and two dorsalis pedis BP measurements and an ABI for each leg was calculated. An ABI ≤ 0.90 was considered abnormal, whereas a value ≥ 1.40 suggested arterial calcification [1].

Other variables obtained during this visit were age, sex, history of arterial hypertension, weight, height, body mass index (BMI) and arm and ankle circumference.

### Analysis

Sample size was calculated to detect a significant kappa index of 0.25 with a bilateral alpha risk of 0.05. The estimated proportion of an abnormal ABI (≤ 0.90) in the diabetic population was 15.6%, of a normal ABI (0.91–1.39) 77% and for ABI values suggestive of calcified arteries (≥ 1.40) 7.4%.

Therefore, a sample size of 200 patients allowed to detect significant differences in the brachial SBP of 2 mmHg with a SD of 10 mmHg (α = 0.05 and β = 0.20).

For the comparison of two quantitative variables, the Intraclass Correlation Coefficient (ICC) was used to evaluate the agreement between the brachial SBP values determined with Doppler and mercury sphygmomanometer and with Doppler and the OMRON monitor.

The scale applied to evaluate the degree of agreement with this coefficient was as follows: <0.10 null agreement, from 0.10 to 0.30 bad agreement, from 0.31 to 0.50 poor agreement, from 0.51 to 0.70 moderate, from 0.71 to 0.90 good and > 0.90 very good agreement. Similarly, the ICC was used to evaluate the agreement between lower limb SBP measurements done with the mercury sphygmomanometer and the OMRON device and between the individual values of ABI determined with both methods.

The weighted kappa index (k) was used to evaluate the agreement between the qualitative ABI values determined with both methods. In this case, the scale used to assess the degree of agreement was: k ≤ 0.20 poor, k from 0.21 to 0.40 weak, from 0.41 to 0.60 moderate, from 0.61 to 0.80 good and k from 0.81 to 1 very good.

Sensitivity, specificity, positive predictive value (PPV), negative predictive value (NPV), positive likelihood ratio and negative likelihood ratio of the OMRON equipment to detect an abnormal ABI (≤0.90) were calculated with the standard formulae.

The differences between the mercury sphygmomanometer and the OMRON monitor for ABI quantitative values are shown in the Bland and Altman plot. The mercury sphygmomanometer was considered to be the gold standard. All analyses were performed using Stata/SE Version 11 (StataCorp, Collage Station, TX, USA).

## Results

### General data

The study included 211 patients with type 2 diabetes. The two lower limbs of each patient were considered independent, thus a total of 421 measurements (one patient had a metatarsal amputation) were carried out.

Patient description is shown in Table 1. The mean age of the participants was 67 years (SD = 10), 51.7% were women and 47.6% (98) had a BMI > 30 Kg/m^2^. Diagnosis of arterial hypertension was reported in 74.1% (149) of patients, 17.1% were smokers and the mean time to diagnose diabetes was 7.1 years (SD = 4,4). Four patients were excluded because of an ankle circumference > 40 cm, they were men, obese and hypertensive, with an average age of 56 years (SD = 5.8).

**Table 1:** Characteristics of the patients studied

**Characteristics**	**Patients (n = 211)**
**Age**, mean (SD)	67 (10)
**Gender**, n (%)	
Male	102 (48.3)
Female	109 (51.7)
**Weight** (Kg), mean (SD)	77 (14)
**Height** (cm), mean (SD)	159 (10)
**BMI** (Kg/m^2^), mean (SD)	30.3 (5.2)
Normal (20–25), n (%)	41 (19.9)
Overweight (26–30), n (%)	67 (32.5)
Obese (>30), n (%)	98 (47.6)
**History of hypertension**, n (%)	149 (74.1)
**Upper arm circumference** (cm), mean (SD)	29 (3)
> 32 cm, n (%)	24 (12.1)
**Ankle circumference **^1^ (cm), mean (SD)	25 (4)
> 32 cm, n (%)	7 (3.5)

SD: Standard Deviation; BMI: Body Mass Index.

### Systolic Blood Pressure (SBP) and Ankle-Brachial Index (ABI)

Table 2 shows the mean values of brachial and ankle SBP and ABI values obtained with the mercury sphygmomanometer and the OMRON HEM-907 monitor. According to the mercury sphygmomanometer abnormal ABI was observed in 40 limbs (9.5%) and 41 (9.7%) were suggestive of calcification. More men than women obtained abnormal ABI values (17.7% *vs*. 1.8%; p < 0.001).

**Table 2:** Systolic blood pressure and ankle-brachial index using the mercury and the electronic device sphygmomanometers

	**Limbs = 421**	
	**Mercury**	**Electronic device**
**Brachial SBP** (mmHg), mean (SD)	140 (17)	140 (17)
**Dorsalis pedis SBP** (mmHg), mean (SD)	158 (30)	157 (30)
**Posterior tibial SBP** (mmHg), mean (SD)	160 (31)	160 (30)
**ABI**, n (%)		
Normal (0.91–1.39)	340 (80.8)	344 (81.7)
Abnormal (≤ 0.90)	40 (9.5)	37 (8.8)
Calcification (≥ 1.40)	41 (9.7)	40 (9.5)
Abnormal (≤ 0.70)	14 (3.3)	12 (2.8)

SD: Standard Deviation; SBP: Systolic Blood Pressure; ABI: Ankle-Brachial Index.

### Agreement on SBP between the mercury sphygmomanometer and the OMRON HEM-907 BP monitor

A very good agreement was found between brachial SBP measured with a mercury sphygmomanometer and brachial SBP measured with the OMRON HEM-907 monitor (ICC = 0.91; CI 95%: [0.87; 0.93]). Similar positive results were found when measuring SBP in the ankle (dorsalis pedis SBP: ICC = 0.85; CI 95%: [0.79; 0.89] and posterior tibial SBP: ICC = 0.89; CI 95%: [0.87; 0.91]).

The mean difference between the two techniques was 0.08 mmHg (SD = 7.4) for brachial SBP. The mean differences in ankle SBP measurements were 0 mmHg (SD = 16.5) in the dorsalis pedis SBP and -0.28 mmHg (SD = 14.4) for the posterior tibial SBP.

**Figure 1:**
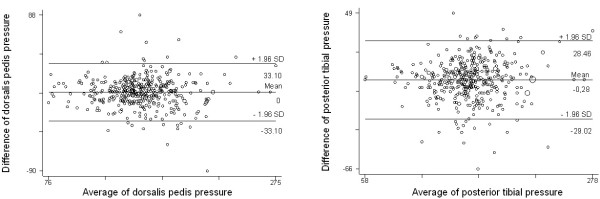
Bland & Altman graphic: differences between mercury sphygmomanometer and OMRON HEM-907 device for ankle systolic blood pressure.

The differences between both techniques were correlated throughout the range of ankle SBP measurements. The rise in SBP did not increase these differences. In the case of the dorsalis pedis SBP, 95% of the differences observed concerning the two techniques were between -33.10 mmHg and 33.10 mmHg. For the posterior tibial artery SBP 95% of the differences were between -29.02 mmHg and 28.46 mmHg (Figure 1).

**Figure 2:**
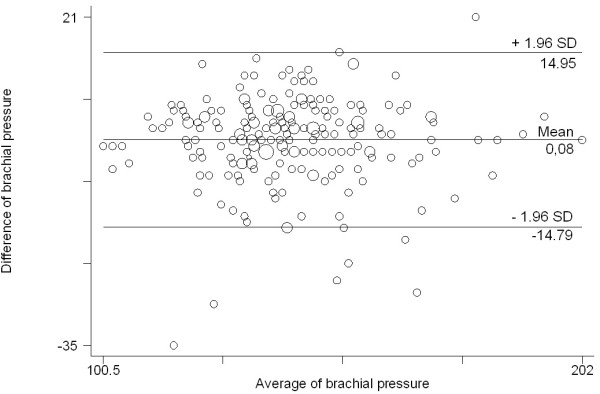
Bland & Altman graphics: differences between mercury sphygmomanometer and OMRON HEM-907 device for brachial systolic blood pressure.

Brachial SBP differences were also correlated throughout SBP values and 95% of the differences in measurements with the mercury sphygmomanometer compared with the OMRON HEM-907 monitor ranged between -14.79 mmHg and 14.95 mmHg (Figure 2).

### Agreement on ABI between the mercury sphygmomanometer and the OMRON HEM-907 BP monitor

**Figure 3:**
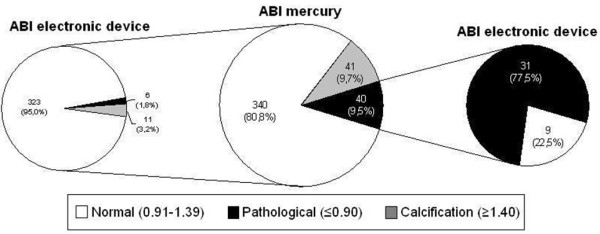
Ankle-brachial index (ABI) measured with the mercury and the electronic device sphygmomanometers.

From the 40 limbs with an abnormal ABI measured with the mercury sphygmomanometer, 9 (22.5%) obtained a normal value with the OMRON monitor and none presented values of calcification (Figure 3). On the other hand, six measurements carried out in lower limbs had criteria of PAD and 11 of calcification with the OMRON device and obtained normal values with the mercury sphygmomanometer. Therefore, 5% of the lower limbs considered normal by the mercury sphygmomanometer were abnormal or calcified according to the OMRON monitor. Modifying the cut off value for the ABI to ≤ 0.70 [18], the OMRON sphygmomanometer only missed diagnosis of PAD in two legs.

The agreement between ABI values measured with the mercury and the OMRON sphygmomanometers was good (weighted k =0.68; CI 95%: [0.55; 0.79]) (Table 3).

**Table 3:** Indices that assess the quality of the diagnostic test

Weighted* Kappa (95% CI)	
ABI ≤ 0.90	0.68 (0.55–0.79)
ABI ≤ 0.70	0.92 (0.81–1.00)
**Sensitivity (95% CI)**	
ABI ≤ 0.90	77.5% (61.5–89.2)
ABI ≤ 0.70	85.7% (57.2–98.2)
**Specificity (95% CI)**	
ABI ≤ 0.90	98.2% (96.1–99.3)
ABI ≤ 0.70	100% (99.0–100)
**PPV (95% CI)**	
ABI ≤ 0.90	83.8% (68.0–93.8)
ABI ≤ 0.70	100% (73.5–100)
**NPV (95% CI)**	
ABI ≤ 0.90	97.3% (94.9–98.8)
ABI ≤ 0.70	99.4% (98.0–99.9)
**Positive Likelihood ratio**	
ABI ≤ 0.90	42.5 (18.9–95.5)
**Negative Likelihood ratio**	
ABI ≤ 0.90	0.3 (0.1–0.4)

BP measurement using the mercury sphygmomanometer was considered the reference method.

ABI: Ankle-Brachial Index; PPV: Positive Predictive Value; NPV: Negative Predictive Value.

*Quadratics weights.

Sensitivity and specificity were 77.5% and 98.2%, respectively, with a PPV of 83.8% and a NPV of 97.3%. The agreement increased with ABI cut-off ≤ 0.70 (weighted k = 0.92; CI 95%: [0.81; 1.00]). This cut-off improved also sensitivity and specificity to 85.7% and 100%, respectively, as well as the PPV and NPV that reached 100% and 99.4%, respectively.

**Figure 4:**
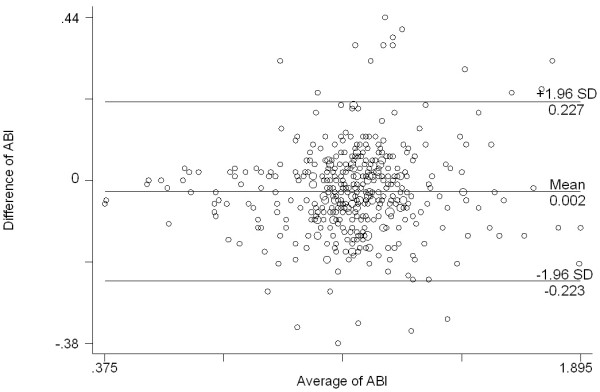
Bland & Altman graphics: differences between individual values of ABI measured with the mercury and the electronic device sphygmomanometers.

The Bland and Altman graph (Figure 4) showed that the differences between the two techniques were constant for the whole range of ABI values and these differences did not change increasing the ABI value. Ninety-five percent of the differences in ABI observed between the two techniques were between -0.223 mmHg and 0.227 mmHg corresponding to the interval represented in Figure 4. In addition, a good agreement was found between individual values of ABI measured with a mercury sphygmomanometer and with the OMRON HEM-907 monitor (ICC = 0.86; CI 95%: [0.81; 0.90]).

## Discussion

The results of this study show good agreement between ABI values obtained with the mercury sphygmomanometer compared to those measured with the OMRON BP monitor. The sensitivity and specificity of the ABI measured with the OMRON HEM-907 sphygmomanometer were 77.5% and 98.2%, respectively with a PPV of 83.8% and a NPV of 97.3%.

When the cut-off value is defined to ≤ 0,70 sensitivity and specificity improve to 85.7% and 100% respectively. Moreover PPV and NPV reach 100% and 99.4%. Only in two of 14 lower limbs with ABI ≤ 0.70 measured with the mercury sphygmomanometer did the OMRON monitor obtain normal ABI values. That could be explained by the fact that the reliability of the examination improves as lower are the ABI values obtained [19].

Leng et al. [18] did a study with 1592 patients and observed that the ones that presented at baseline ABI ≤ 0,90 showed moderate specificity (83,2%), and a likelihood ratio of 1.76 in predicting fatal and non-fatal cardiovascular events after five years. However a lower index (ABI ≤ 0,70) showed also better specificity (95.4%) and a higher likelihood ratio (3.07).

Differences were observed between SBP measured with Doppler and the OMRON BP monitor and SBP obtained with Doppler and the mercury sphygmomanometer, particularly in the lower limbs. Arterial stiffness is more prevalent in the arteries of the lower limbs, particularly in diabetic and elderly patients, which may account for the distortion of SBP values and the better correlation between the two methods found in the upper limbs [20].

This special feature of the lower limbs makes difficult to find an appropriate instrument to replace the mercury sphygmomanometers to measure ABI. The mercury sphygmomanometer is considered to be the gold standard but its removal based on environmental reasons makes necessary to find an equivalent replacement.

Similarly to the mercury BP monitors, aneroid sphygmomanometers use auscultation of Korotkoff sounds to determine SBP. However, the need for frequent calibration and the possibility of undetected failures render aneroid sphygmomanometers unreliable [21,22].

Kollias et al. [12] performed a study with 94 patients and found a strong correlation between ocillometric and Doppler ABI (r 0,80). However patients with arterial calcification diagnosed by Doppler were excluded from the beginning.

In several publications oscillometric sphygmomanometers have shown very low sensitivity in ABI measurements [9-11]. Metanalisis [23] of studies that compare ABI obtained with automated oscillometric devices and the conventional doppler method attain sensibility of 69 ± 6% and specificity of 96 ± 1% to diagnose PAD. But these studies present great heterogeneity both with the devices and the methodology.

In the automated oscillometric devices systolic and diastolic blood pressure is indirectly calculated with an algorithm that can be affected by factors other than blood pressure, mainly arterial stiffness [8]. Arterial calcification increases with age and diseases such as diabetes mellitus. PAD is highly prevalent in diabetes patients [1,2] and therefore it is important to determine a reliable ABI. This is a great inconvenience because there are automated devices in the market very comfortable and fast to determine blood pressure in the arms and ankles which could be very useful in clinical practice. Hybrid sphygmomanometers combine the advantages of auscultatory and automated BP monitors. Although the technique is more complicated than the automated ones, they reduce the possibility of error by automatically inflating and deflating the cuff and facilitating the measurements.

A limitation of this study is the type of patients selected. The chances of error increase in a sample of elderly diabetic patients with an obesity rate of 47.6% [24,25]. However, it has also been an opportunity to test these two methods in more challenging conditions than the everyday clinical practice.

## Conclusions

The results of the current study show that ABI measurements using a hybrid OMRON device are comparable to those obtained with the classical mercury sphygmomanometer. As recommended for mercury sphygmomanometers, BP measurements with hybrid monitors should be repeated in patients with ABI values around 0.90 [19], and particularly in patients with ABI values ≥ 0.70.

In conclusion, we propose the hybrid OMRON HEM-907 BP monitor as the replacement of mercury sphygmomanometers in ABI measurement.

### Ethical approval

The study protocol has received institutional review board approval (IDIAP Jordi Gol Ethical Clinical Committee).

## Competing interests

The authors declare that they have no competing interests.

## Authors’ contributions

MB participated in the original research idea, design of the study, statistical analysis, interpretation of data and coordination. MU, JLL, RF participated in de design and the field work. LM participated in de design, statistical analysis and interpretation of data and helped to draft the manuscript. PT participated in the design and helped to draft the manuscript. All authors read and approved the final manuscript.

## Pre-publication history

The pre-publication history for this paper can be accessed here:

http://www.biomedcentral.com/1471-2261/13/15/prepub
